# An R package for simulating growth and organic wastage in aquaculture farms in response to environmental conditions and husbandry practices

**DOI:** 10.1371/journal.pone.0195732

**Published:** 2018-05-03

**Authors:** Damiano Baldan, Erika Maria Diletta Porporato, Roberto Pastres, Daniele Brigolin

**Affiliations:** 1 Bluefarm S.r.l., Venezia Marghera, Italy; 2 Department of Environmental Sciences, Informatics and Statistics, Ca’ Foscari University of Venice, Venezia Mestre, Italy; Universidade de Vigo, SPAIN

## Abstract

A new R software package, RAC, is presented. RAC allows to simulate the rearing cycle of 4 species, finfish and shellfish, highly important in terms of production in the Mediterranean Sea. The package works both at the scale of the individual and of the farmed population. Mathematical models included in RAC were all validated in previous works, and account for growth and metabolism, based on input data characterizing the forcing functions—water temperature, and food quality/quantity. The package provides a demo dataset of forcings for each species, as well as a typical set of husbandry parameters for Mediterranean conditions. The present work illustrates RAC main features, and its current capabilities/limitations. Three test cases are presented as a proof of concept of RAC applicability, and to demonstrate its potential for integrating different open products nowadays provided by remote sensing and operational oceanography.

## Introduction

Virtual technologies are increasingly perceived as a resource for aquaculture science-based management [1]. The selection of sites/areas plays a key role in supporting the sustainable development of this industry within the framework of the Ecosystem Approach to Aquaculture (EAA) [2]. With this respect, identification of Allocated Zones for Aquaculture (AZAs), the selection of individual sites, and the design of Aquaculture Management Areas (AMAs) are three complex, and inter-related issues [3,4]. This task is further complicated by the forecasted long-term trends in environmental parameters, induced by climate changes, which will need to be included in the planning, in order to attempt a sound adaptive management of these activities [5,6]. To face these challenges, nowadays, aquaculture models can largely benefit from the information provided by remote sensing and operational oceanography [7,8,9]. Current efforts are devoted at implementing existing aquaculture models in accessible formats, available to users with different programming expertise. This is expected to help promoting the use of models in aquaculture, and improve the reproducibility of results [10].

Here we describe the new R software package RAC (R package for AquaCulture), which focuses on 4 species, finfish and shellfish, highly important in terms of production in the Mediterranean Sea [11]. RAC simulates the rearing cycle of the European seabass (*Dicentrarchus labrax*), Gilthead seabream (*Sparus aurata*), Manila clam (*Ruditapes philippinarum*) and Mediterranean mussel (*Mytilus galloprovincialis*), both at the individual and the population level. These mathematical models account for growth and metabolism of the individual, based on input data characterizing the forcing functions—water temperature, and food quality and quantity. For finfish, seabass and seabream, food availability is represented by the feed provided by the farmer, while shellfish models require a characterization of quantity and quality of the suspended organic matter. The package provides a demo dataset of forcings for each species, as well as a typical set of husbandry parameters for Mediterranean conditions, in order to allow the user to run a default simulation. The present work illustrates the main features of RAC and delineates its current capabilities in terms of predicting growth and environmental interactions of aquaculture. RAC outputs represent a baseline which could be integrated with environmental spatial data, economic-social criteria and policy issues, in order to support the decision makers in the identification of suitable area for aquaculture activities and for the implementation of an EAA [2,9]. Three test cases were carried out in the Adriatic Sea (Italy) as a proof of concept of RAC applicability: a) individual model runs for each of the 4 species: seabass, seabream, clam and mussel; b) evaluation of site suitability for mussel aquaculture; c) evaluation of the response of seabream growth and organic waste release to climate-induced changes in water temperature.

## Software characteristics and capabilities

RAC simulates the rearing cycle of the four farmed species, both at the individual and the population level. The package is based on a set of models independently validated in previous studies. RAC is released as open-source and can be freely downloaded from its website, https://cran.r-project.org/package=RAC. This section will briefly introduce the models (sections Individual model and Population up-scaling), therefore focusing on input/output information flow (section Inputs and outputs), the package structure (section Package structure), and the instructions required for running it (section Instructions).

### Individual model

The growth of the individual is simulated by solving the following general energy balance equation:

$$\frac{dw}{dt}=\frac{A-C}{\varepsilon }$$ (1)

where:

- w is the wet weight [g]
- t is time [d]
- A is the anabolic rate [J d^-1^]
- C is the catabolic rate [J d^-1^]
- ε [J g^-1^] represents the energy density of body tissues.

The weight increment is therefore described as the difference between the anabolism and the catabolism. Formulations and parameters are reported in the Supporting Information (Tables in S1 and S2 Files). These terms are species-specific and are described in detail in Brigolin et al. [12] (Mediterranean mussel), in which Eq (1) is modified in order to account for reproduction, Solidoro et al. [13] (Manila clam), Brigolin et al. [14] (gilthead seabream), Brigolin et al. [15] (European seabass).

### Population up-scaling

According to the methodology used by Bacher and Gangnery [16], the individual model was up-scaled to the population level by means of a set of Monte Carlo simulations, which were used for estimating the size structure of the population (see [12,15]). Such differences were accounted for by assigning a different initial weight and maximum clearance rate to each shellfish specimen, and a different initial weight and ingestion rate to each finfish specimen, in order to reflect the variability in individual phenotypes, as well as the differences in the localization of specimens within the farm (for shellfish). Individuals in the population have a fixed mortality rate, forced to be discontinuous by stocking and harvesting of animals in the farm.

### Inputs and outputs

RAC inputs and outputs, summarized in Tables 1 and 2, are species specific. Finfish are forced by the time series of water temperature (°C), feed availability (g d^-1^), and feed composition (relative %), this latter one characterized in terms of proteins, lipids and carbohydrates, and not dependent on time. Mussels are forced by daily time series of water temperature (°C), chlorophyll-a concentration (μg l^-1^), Particulate Organic Carbon concentration (POC, mg C l^-1^), Particulate Organic Matter concentration (POM, mg C l^-1^), Total Suspended Matter concentration (TSM, mg C l^-1^) and the POC characterization in terms of C/P and N/P molar ratios (-). The Clam model is forced by the water temperature (°C) and the chlorophyll-a concentration (μg l^-1^).

**Table 1:** Forcing variables required as input data for each model species at both individual and population level.

		Forcings required							
	Model species	Feeding rate + Feed composition	SST	CHL-*a*	TSM	POM	POC	POC C/P	POC N/P
**Individual**	Mussel		√	√	√	√	√	√	√
	Clam		√	√					
	Seabass	√	√						
	Seabream	√	√						
**Population**	Mussel		√	√	√	√	√	√	√
	Clam		√	√					
	Seabass	√	√						
	Seabream	√	√						

**Table 2:** Output data obtained from the models of each species at both individual and population level.

		Output data												
	Model species	W	L		Exc	Wst	Ing	T fun	MR	Pf	CNP	O_2_	NH_4_	N
**Individual**	Mussel	√	√		√			√	√	√	√	√	√	
	Clam	√	√					√	√					
	Seabass	√			√	√	√	√	√			√	√	
	Seabream	√			√	√	√	√	√			√	√	
**Population**	Mussel	√	√		√			√	√	√	√	√	√	√
	Clam	√	√					√	√					√
	Seabass	√			√	√	√	√	√			√	√	√
	Seabream	√			√	√	√	√	√			√	√	√

W, weight; L, length; Exc, excretion; Wst, Waste produced; T fun, Temperature response function; MR, Metabolic rates; Pf, Pseudofaeces; CNP, C:N:P composition; O_2_, O_2_ consumed; NH_4_, NH_4_ produced; N, Number of individuals.

RAC simulations generate two main classes of outputs: i) vector outputs, providing a time series of the number of individuals in the farmed population; ii) matrix outputs, with the mean and the standard deviation of each model state variable, as well as metabolic processes, which are estimated over the distribution of simulated individuals. These output variables, which are different for the 4 models, are summarized in Table 2. Finfish models compute the weight of the individual (g), the ingestion rate (g d^-1^), temperature response functions [–], metabolic rates (anabolism and catabolism, J d^-1^), faeces produced (in terms of proteins, lipids and carbohydrates, g d^-1^ for individuals or kg d^-1^ for population), uneaten feed (in terms of proteins, lipids and carbohydrates, g d^-1^ for individuals or kg d^-1^ for population), O_2_ consumption (g d^-1^ for individuals or kg d^-1^ for population) and ammonia production (gN d^-1^ for individuals or kgN d^-1^ for population). Mussel model computes the somatic and gonadic dry weight of the individual (g), the wet weight of soft tissues (g), and the total weight including the shell (g), the length (cm), faeces and pseudofaeces produced (in terms of C, N and P; g d^-1^ for individuals or kg d^-1^ for population), metabolic rates (anabolism and catabolism J d^-1^), CNP content of animal tissues (g), temperature limitation function, O_2_ consumption (g d^-1^ for individuals or kg d^-1^ for population) and ammonia production (gN d^-1^ for individuals or kgN d^-1^ for population). Clam model outputs include the wet weight of the individual (g), temperature limitation function, metabolic rates (J d^-1^), and shell length (mm). The number of individuals is also provided as an output. Finally, the number of days needed to reach the commercial size from the beginning of the integration period is reported for all the models.

### Package structure

**Fig 1:**
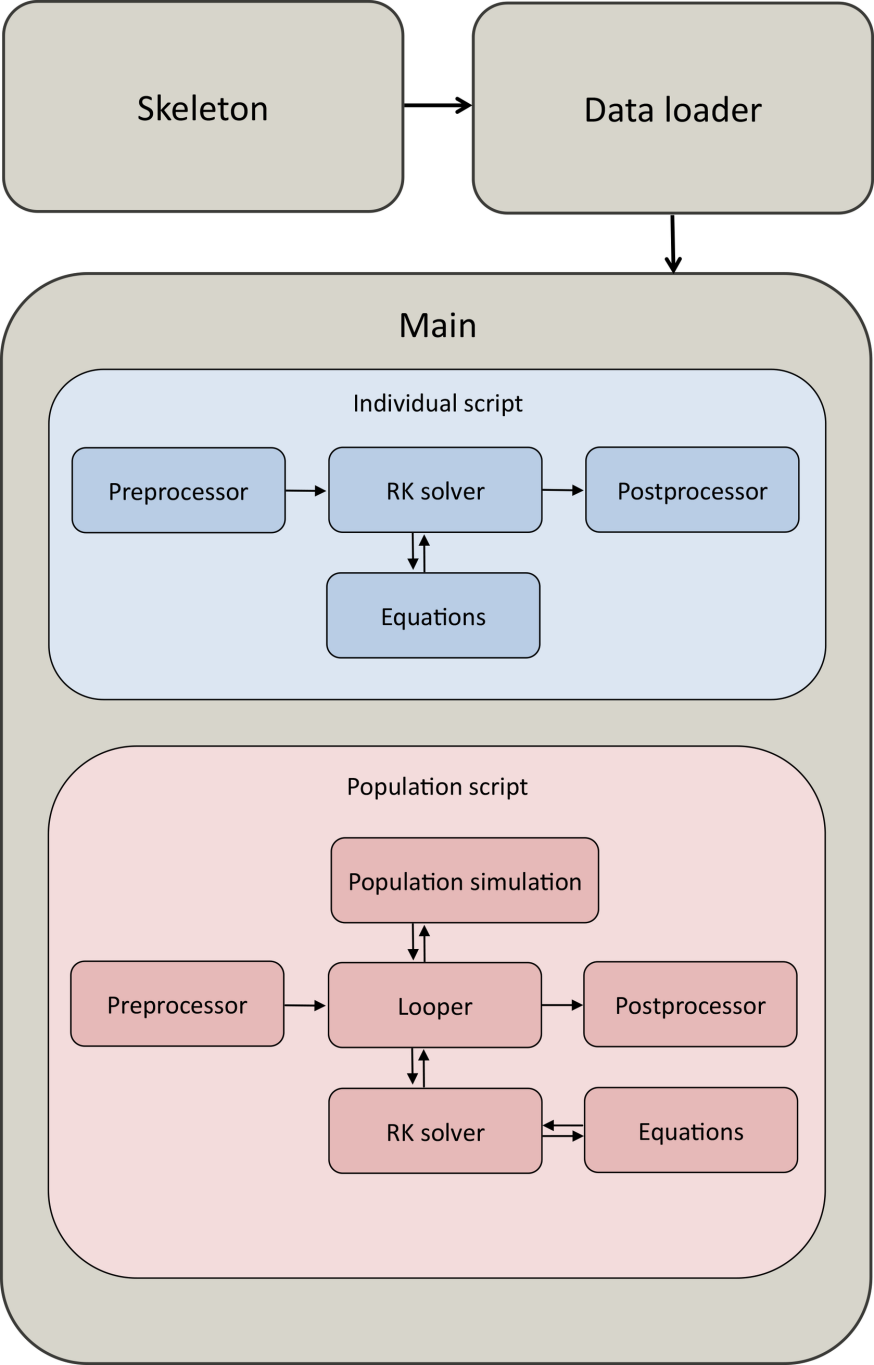
General workflow of RAC package. Arrows show the information flow. The grey boxes indicate the functions called directly by the user.

The general workflow of RAC is schematically described in Fig 1. SKELETON function creates a folder structure at a user defined path and pastes to the structure pre-formatted input files, which can be modified by the user. The subsequent execution of this function overwrites all the files, if present. DATA-LOADER function loads the input data located in the folders to the workspace, interpolating them, in order to fill those parts of the data series that are unequally spaced, thus meeting the requirements of the main script. PRE-PROCESSOR function converts the forcing units (*e*.*g*.: computes detritus + zooplankton C concentration as the difference between the POC concentration and phytoplanktonic C concentration). Additionally, the pre-processor function plots the interpolated forcing values, saving them into the previously created folder structure. The pre-processor can be executed by the main script as many times as required in order to satisfy the user requirements. Subsequently, MAIN function calls all the functions that are required to solve the balance and to save the results. The RK-SOLVER function implements the 4^th^ order Runge-Kutta scheme for the solution of the bioenergetics balance. Equations describing limitation terms, and anabolic and metabolic rates are contained in the EQUATIONS function, called by the RK-SOLVER function. Outputs of the RK-SOLVER are processed by the POSTPROCESSOR function that plots and saves them in files. The population script contains also the LOOP function that runs the Monte Carlo simulation and computes the statistics of the simulated outputs. Finally, the POPULATION function solves the population equation, based on mortality rate parameters and on husbandry practices.

### Instructions

RAC package requires the subsequent execution of three instructions from the R console. The first instruction creates a directory structure at the path specified by the string variable “userpath”. This instruction populates the directory with a ready to use dataset of forcings and parameters, which can be subsequently modified by the user. The second instruction loads the list of model forcings into the R workspace contained at the user defined path. The third instruction runs the model with the loaded forcings and saves the textual and graphic results into the directories specified by the string “userpath”. In order to select the species to model, and the level of hierarchical organization (individual or population), two parts of the string need to be specified by the user, while the third part reports the function of interest. In the first part of the string, the user indicates the species (Bream, Bass, Mussel, Clam), while in the second part indicates “_ind_” or “_pop_”, in order to run the individual or the population model respectively.

As an example, in order to run the Gilthead seabream individual model, the following instructions need to be specified:

**Bream_ind_skeleton (userpath)**

**forcings <–Bream_ind_dataloader (userpath)**

**Output <–Bream_ind_main (userpath, forcings)**

On the other hand, running the Mussel population model will require the following instructions:

**Mussel_pop_skeleton (userpath)**

**forcings <–Mussel_pop_dataloader (userpath)**

**Output <–Mussel_pop_main (userpath, forcings)**

## Case studies

**Fig 2:**
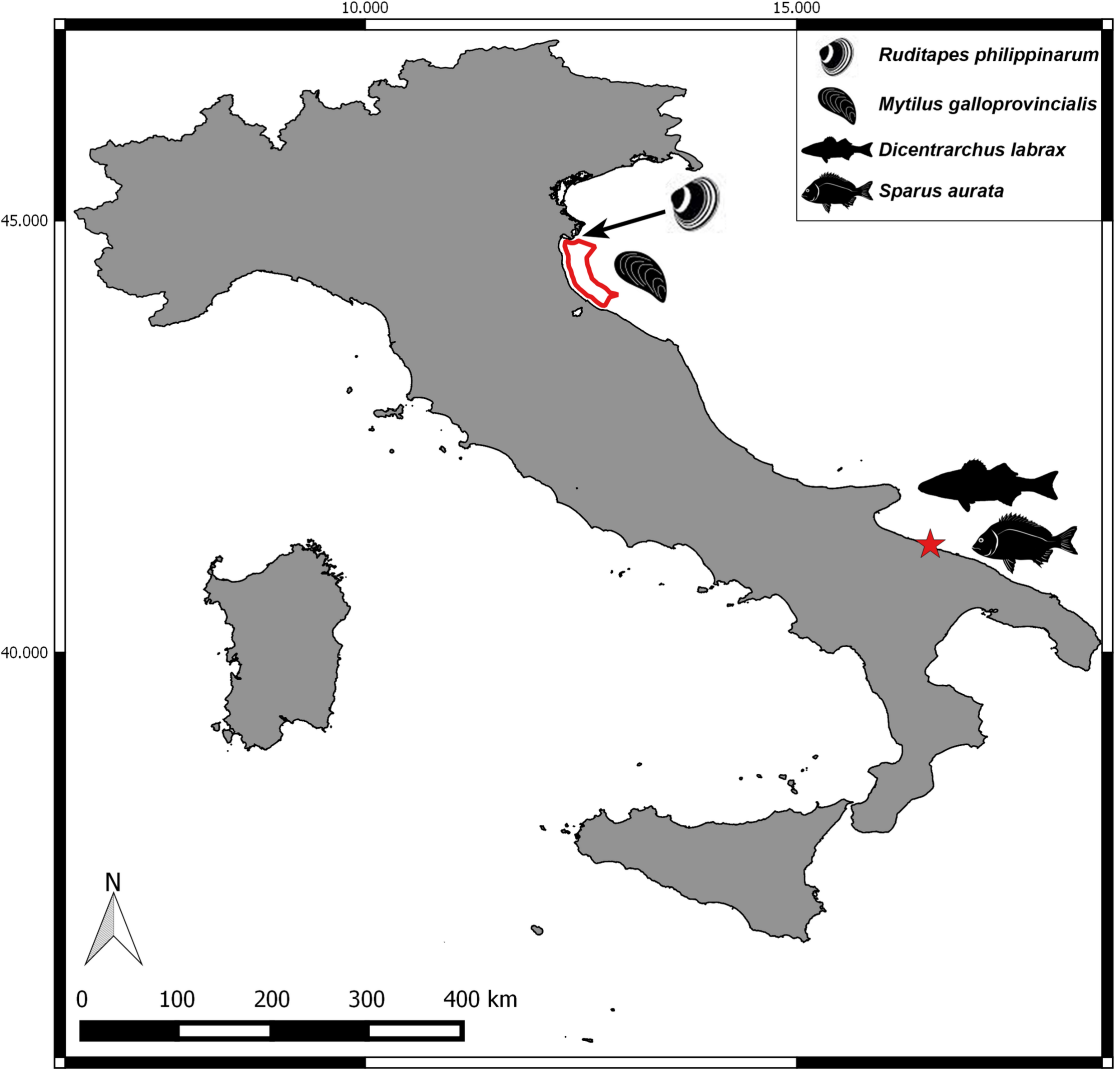
Case study sites and species. *M*. *galloprovincialis* and *R*. *philippinarum*, and finfish, *S*. *aurata* and *D*. *labrax*. The shapefile of Italian boundaries was downloaded from DIVA-GIS dataset (freely available at http://www.diva-gis.org/Data) and the layout was made in QGIS version 2.18.5.

In order to demonstrate the applicability of RAC, and the resources potentially provided by operational oceanography and remote sensing data for running the model, the following set of simulations was performed:

1. individual models were run for the 4 species, using as input present environmental data, taken from the sites marked in Fig 2 - model predictions included growth trajectories, and organic matter waste;
2. mussel individual model was run repeatedly over a spatial domain which represents a portion of sea localized in a highly productive coastal area, in the Northern Adriatic Sea, Emilia-Romagna region—model runs were used to estimate the growth performance of the mussel over this area, which was quantified as days required by the mussel for reaching the length of 5 and 7 cm;
3. water temperature time series for 2049–2050, predicted by climate models, were used to run the seabream population model at a site located in the southern Adriatic Sea, Puglia region (see Fig 2). These simulations were aimed at comparing growth, uneaten feed and faeces release expected under different scenarios of global warming. In order to upscale the individual to population model, a number of 5000 runs, each representing one individual with different initial weight, 80 ± 8 g, and ingestion rate, 0.09 ± 0.018 g food g fish^-m^ day^-1^, were run via Monte Carlo simulation. This number of model runs was empirically found to be the minimum needed to stabilize the results [12]. Farm characteristics and husbandry parameters are resumed in Table 3, and were assigned on the basis of the papers by Solidoro et al. [13], Pastres et al. [17] and Brigolin et al. [12,14,15]. Environmental forcings provided to the models are also resumed in Table 3, and details on data sources and processing are provided in the following section.

**Table 3:** Details of environmental variables, input and husbandry parameters used to run individual (Mussel, Clam, Seabass and Seabream) and population (Seabream) models.

	Individual				Population
	Mussel	Clam	Seabass	Seabream	Seabream
**Environmental variables**					
SST	Copernicus, 1km	Copernicus, 1km	Copernicus, 1km	Copernicus, 1km	CNRM CM5 RCP 45 and 85 (Future)
Chlorophyll-a	Copernicus, 1km	Copernicus, 1km	-	-	-
Feed	-	-	[15]	[14]	[14]
TSM	[12,18]	-	-	-	-
POM	[12,18]	-	-	-	-
POC	[12,18]	-	-	-	-
**Input Parameters**					
Seed, g	0.0333944	0.00702	24	80	80 (±8)
Seeding day	05/09/2015	01/03/2015	15/05/2015	01/06/2015	01/06/2049
Harvesting day	05/07/2016	15/06/2016	14/06/2016	01/05/2016	01/05/2050
**Husbandry parameters**					
Seeded individual, n	-	-	-	-	52988
Harvested individual, n	-	-	-	-	52988
Natural mortality rate, d^-1^	-	-	-	-	0.00041
Number of runs, n	-	-	-	-	5000

### Environmental variables

#### Present time data (2015–2016)

The SST and Chlorophyll-*a* (Chl-*a*) data were extracted from the Copernicus—Marine Environment Monitoring Service (http://marine.copernicus.eu/). For the purposes of this study, satellite data level 4 with a spatial resolution of 0.008 (SST) and 0.013 degrees (Chl*-a*) (~1 Km), from 01/03/2015 to 30/09/2016 were downloaded. As previously described, the shellfish models require TSM, POC and POM concentrations as forcing variables but, since satellite TSM, POC and POM were not available for the study area, these values were imposed on the basis of existing data collected at mussel farms in the area [12,18].

#### RAC spatial application

The area selected for running the model is located in the North Adriatic Sea, along the coastline of Emilia-Romagna region, where mussel farming represents an important activity (21.6 10^3^ metric tons in 2013, 33.6% of the national production [19]), and aquaculture zoning and site-selection in the continental shelf comprised between 3 and 12 nm recently received increasing attention [9]. Model application in this area allowed to derive an indicator of potential growth, selected on the basis of its capability to be easily communicated to stakeholders, which is the number of days required to reach the minimum size required by the law for commercialization—5 cm shell length for mussels. In this work, we have also evaluated the days required to reach 7cm, as this represents the ideal commercial target size to be achieved.

In order to apply the model, environmental data were extracted from NetCDF (Network Common Data Form) datasets, and converted in data matrices (spatial coordinates + daily values). Hence, the forcing variables time series at each grid cell were used as input for the RAC model functions. RAC outputs were stored in a matrix, and associated to the cell spatial coordinates. Two maps of the number of days required to reach both sizes for commercialization were finally created, and the complete set of RAC daily outputs were stored in NetCDF files. R code to run the individual mussel model over a spatial domain is reported in S1 Appendix.

#### Future climate scenarios (2049–2050)

Time series of future SST data were downloaded from the CEDA ESGF data node (https://esgf-index1.ceda.ac.uk/projects/esgf-ceda/) taking advantage of the results obtained within the EURO-CORDEX initiative ([20] Coordinated Regional Climate Downscaling Experiment). This Regional Climate Model is based on the IPCC Fifth Assessment Report (AR5) CMIP5 (Coupled Model Intercomparison Project). In order to highlight the effects of future temperature changes, we have chosen two Representative Concentration Pathways (RCP [21]) scenarios, 4.5 and 8.5 with a spatial resolution of 0.11 degrees (EUR-11; ~12.5 km). We have selected the climatic models ran by the Centre National de Recherches Météorologiques Coupled Global Climate Model, version 5 (CNR-CM5) for the 2049–2050 time period.

## Potential uses and discussion

### Individual model outputs

**Fig 3:**
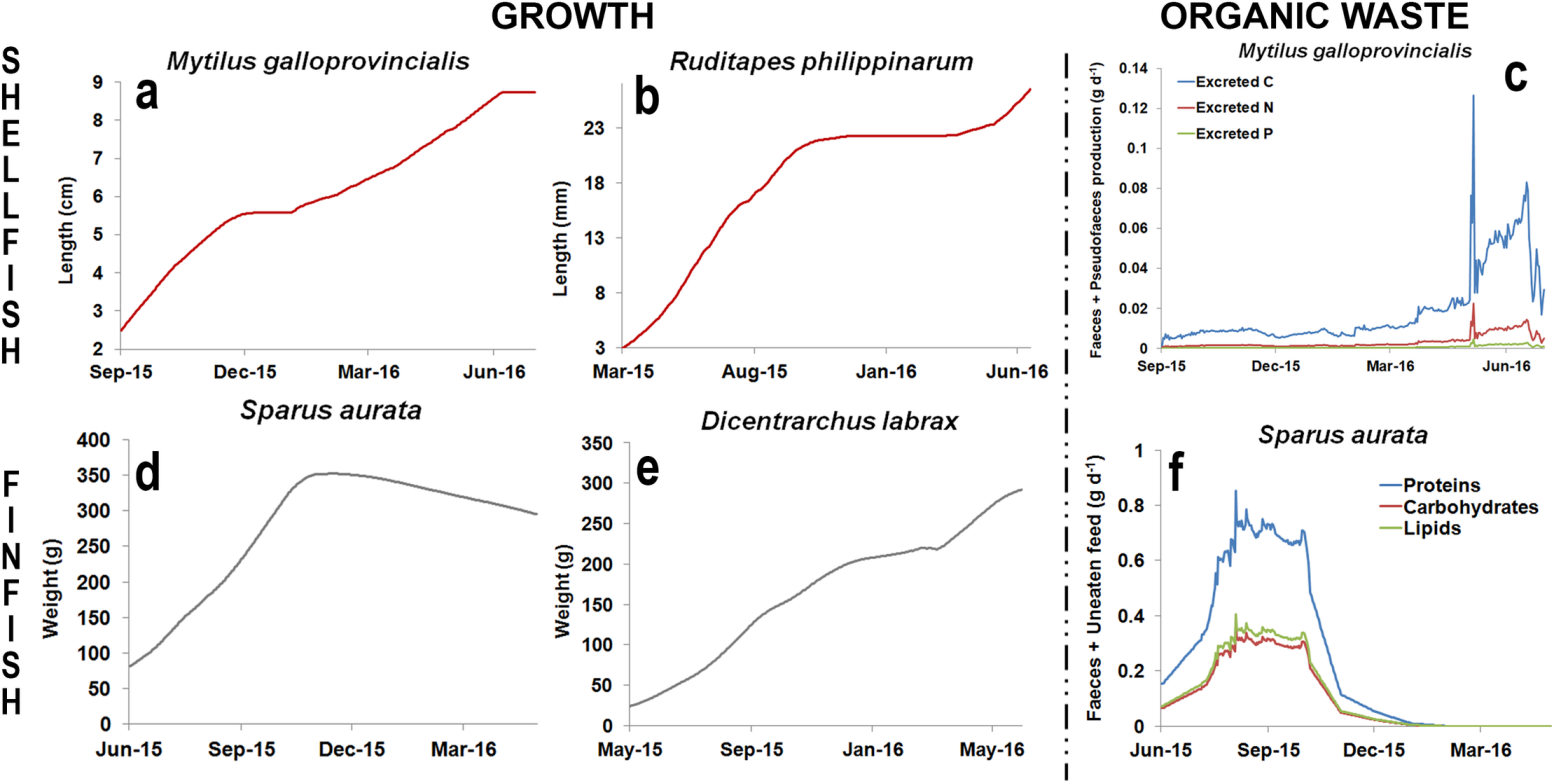
Current individual model outputs of shellfish, *M*. *galloprovincialis* and *R*. *philippinarum*, and finfish, *S*. *aurata* and *D*. *labrax*. On the left panels the individual growth in terms of length (cm and mm) for the shellfish (a and b) and of weight (g) for the finfish (d and e). On the right panel the organic waste produced by *M*. *galloprovincialis* (c) in terms of faeces and pseudofaeces (g d^-1^) and the quantity of uneaten feed (g d^-1^), in terms of protein, lipids and carbohydrates, produced by *S*. *aurata* (f).

Fig 3A and 3B show the growth in length for the shellfish species (mussel and clam), while Fig 3D and 3E show the growth in weight for the finfish species (seabream and seabass). For all the species, the individual growth predicted by the models under the effect of environmental forcings provided by operational oceanography products is in good agreement with the results obtained in previous studies in which model validation was carried out [12,13,14,15,17]. Fig 3C and 3F show the model output in terms of organic waste, which is represented by faeces and pseudofaeces produced per individual mussel (g d^-1^), and the sum of faeces produced and uneaten feed per individual seabream (g d^-1^). For mussels, predicted values are in agreement with the measurements performed in eastern Canada [22], with faeces ranging between 29.1 and 44.4 mg ind.^-1^ d^-1^, and for seabream simulations agree with the results published for the Southern Adriatic Sea [14], with values reaching 1.8 g ind^-1^ d^-1^.

### Space suitability for mussel farming: Time to reach the market size

**Fig 4:**
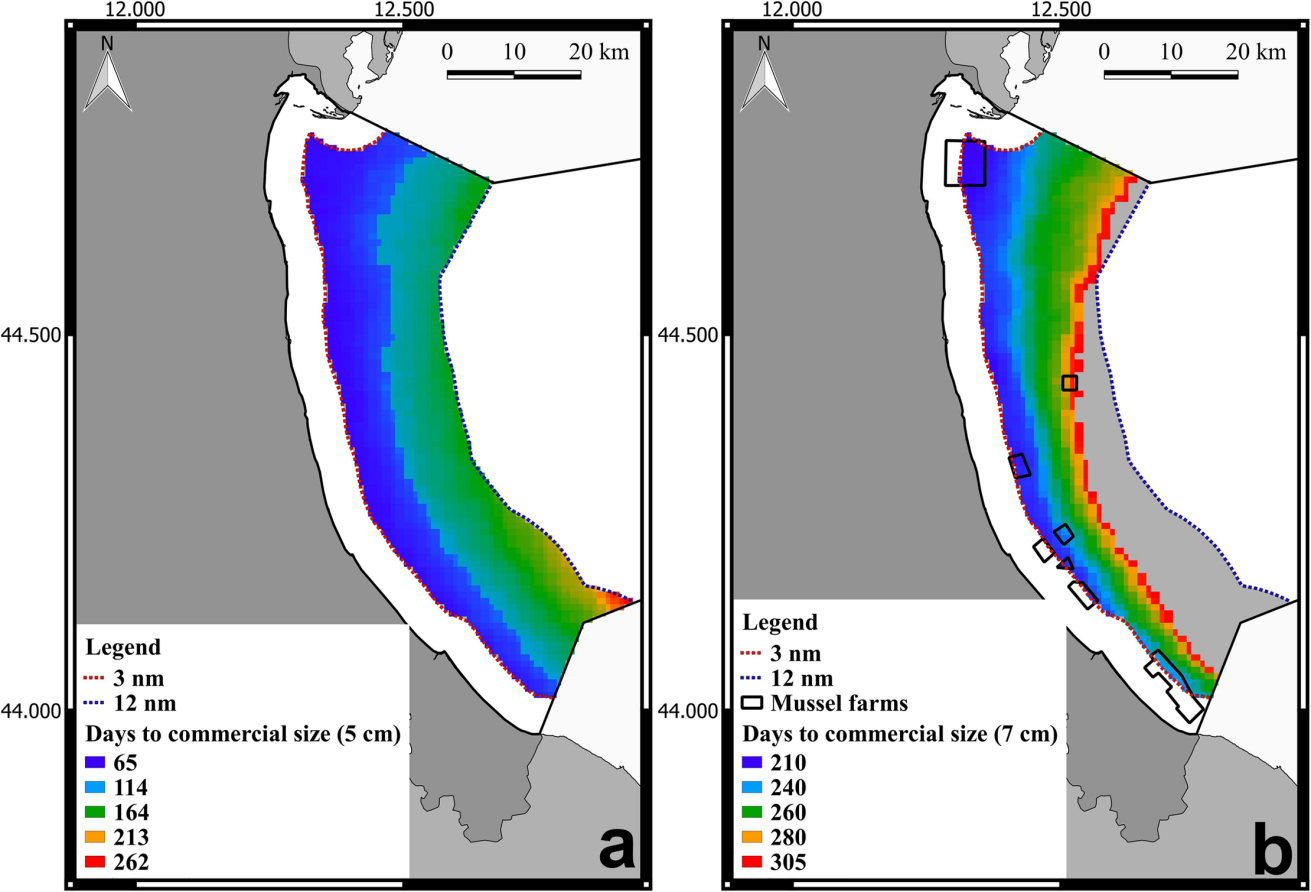
RAC spatial application: Output of *M*. *galloprovincialis*. In these maps are represented the number of days necessary to reach the commercial size of 5 cm (a) and 7 cm (b). The shapefile of Italian boundaries was downloaded from DIVA-GIS dataset (freely available at http://www.diva-gis.org/Data) and the layout was made in QGIS version 2.18.5.

As one can see in Fig 4A mussels, seeded at 2.5 cm, reach the minimum size for commercialization, 5 cm, in a period comprised between two months and eight months and half. As visible in Fig 4B, mussels reach in the northern area the size of 7 cm in approximately 8 months, while in the south-eastern area this size is not achieved within the simulated period of 10 months. Results suggest that the northern part of this area is more suitable compared to the southern one. Indeed, this portion of sea, being under the direct influence of the Po river plume, is highly rich in nutrients, and sustains an elevated primary productivity [23,24].

### Climate change effects on growth and organic waste load

**Fig 5:**
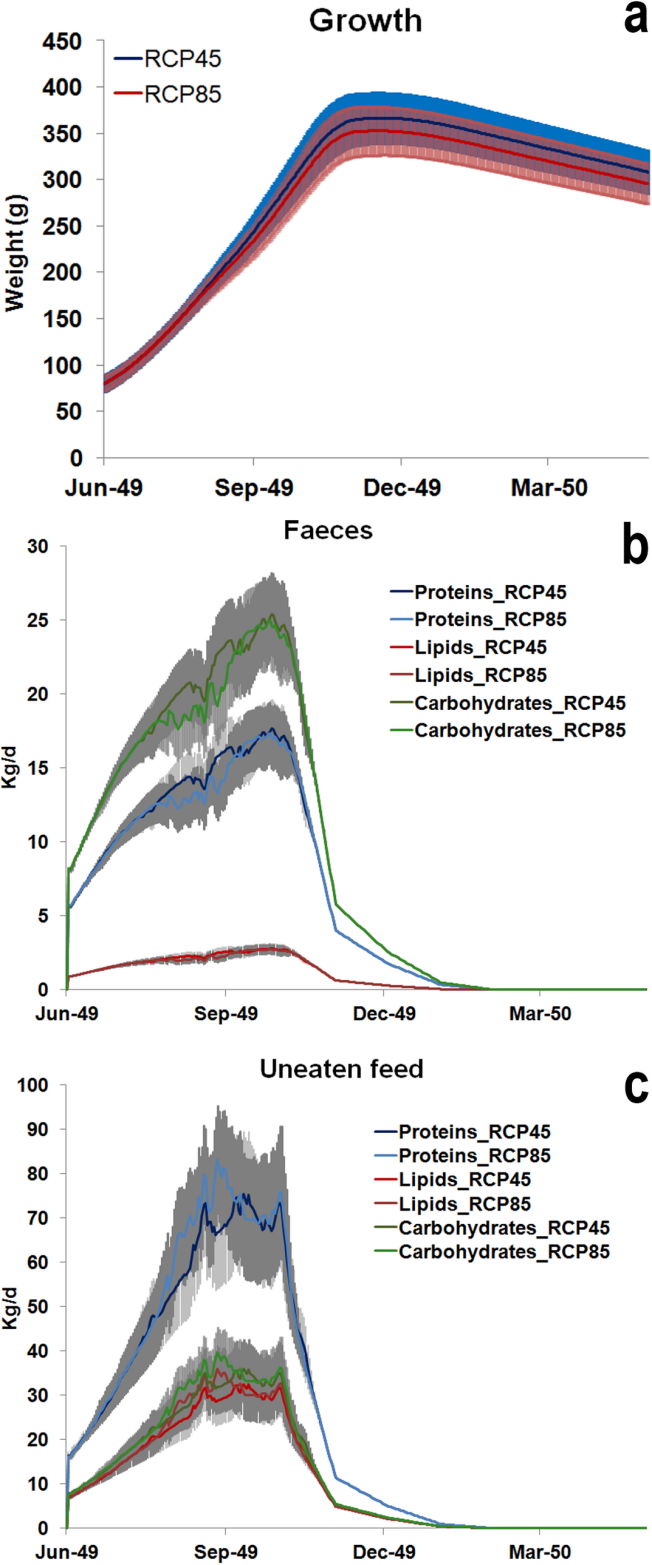
Future population model outputs of *S*. *aurata* simulated at Bisceglie (Southern Adriatic Sea) under RCP 4.5 and 8.5 temperature scenarios. a) mean growth and ± standard deviation (g); b) the uneaten feed in terms of protein, lipids and carbohydrates (Kg d^-1^); c) faeces produced in terms of protein, lipids and carbohydrates (Kg d^-1^).

Fig 5A shows the forecasted effects of water temperature change on seabream growth, including variability in weight trajectories among individuals belonging to the same farmed population. Growth performance differs during the warm season, with the highest values in correspondence to the more “optimistic” scenario, RPC4.5, with respect to RCP8.5. A similar trend for RCP 4.5 and 8.5 is detectable both for faeces, Fig 5B, and uneaten feed, Fig 5C, quantified in terms of proteins, lipids and carbohydrates. It is worth remarking here that the high variability associated to these outputs (standard deviation within the population is shown in grey) poses limitation to the overall significance of differences detected under the two scenarios. The rapid decrease of uneaten feed and faeces, reaching 0 in December, are due to the pause in feeding during winter months, which is present in current days husbandry practices. These husbandry practices may be subjected to substantial variations in the future. To this regard, we remark model results could help in identifying the most appropriate practices, based on the expected environmental changes, and effectively contribute to the planning of sound adaptation strategies for aquaculture.

## Conclusion and next steps

This paper provides a synthetic overview of design and applicability of the RAC package. The reasonableness of RAC estimations was confirmed by the agreement between the results obtained in test cases performed within the present work with previous studies in which models were validated. Runs carried out demonstrate the potential of RAC for integrating different open products nowadays provided by remote sensing and operational oceanography. This goes in the direction of overcoming limitations imposed by data scarcity, recognized as a major obstacle to the application of virtual technologies for aquaculture [1]. Indeed, this tool represents a resource for simulating the rearing cycle of different species, finfish and shellfish, under different scenarios of change of future climate conditions. This has the potential to support a science-based design of aquaculture areas, *e*.*g*. integrating the outputs obtained from this tool with site selection criteria (*i*.*e*.: depth, significant wave height, distance to harbour, etc…) in a multi-criteria evaluation process [9], and contribute to an effective implementation of maritime spatial planning [25]. RAC outputs can guide the implementation of site selection and management, for instance integrating the results with deposition models [15], in order to achieve the sustainable development of aquaculture activities. Future efforts will focus on RAC maintenance and improvement, in response to user feedbacks. One upcoming update will be the integration of a new routine for automating the runs performed in spatial explicit mode, starting from input rasters of environmental data. A further planned update will be embedding in the package a deposition module [15].

## Supporting information

S1 File Model equations. **Table A.** Model state variables, forcings, and functional relationships of *M*. *galloprovincialis*–as in Brigolin et al. (2009).
**Table B.** Functional expressions used in the individual growth models of *D*. *labrax* and *S*. *aurata*–as in Brigolin et al. (2010; 2014).
**Table C.** Model state variables, forcings, and functional relationships of *R*. *philippinarum*—as in Solidoro et al. (2000).
(PDF)

S2 File Model parameters. **Table A.** Parameters used in the *Mytilus galloprovincialis* growth model.
**Table B.** Parameters used in the *Sparus aurata* (SA) and *Dicentrarchus labrax* (DL) growth models.
**Table C.** Parameters used in the *Ruditapes philippinarum* growth model.
(PDF)

S1 Appendix R code to run the individual mussel model spatially explicit. (PDF)

## References

[1] Ferreira JG, Aguilar-Manjarrez J, Bacher C, Black K, Dong SL, Grant J<, et al. Progressing aquaculture through virtual technology and decision-support tools for novel management. In Farming the Waters for People and Food. Ed. by R.P. Subasinghe, J.R. Arthur, D.M. Bartley, S.S. De Silva, M. Halwart, N. Hishamunda, C.V. Mohan, and P. Sorgeloos. Proceedings of the Global Conference on Aquaculture 2010, Phuket, Thailand; 2010 September 22–25. FAO, Rome and NACA, Bangkok; 2012. p. 643–704.

[2] Soto D, Aguilar-Manjarrez J, Brugere C, Angel D, Bailey C, Black K, et al. Applying an ecosystem-based approach to aquaculture: principles, scales and some management measures. In: Soto, D., Aguilar Manjarrez, J., Hishamunda, N. (Eds.), Building an ecosystem approach to aquaculture. FAO/Universitat de les Illes Balears Expert Workshop; Proceedings No. 14; 2007 May 7–11; Palma de Mallorca, Spain. FAO Fisheries and Aquaculture, Rome, FAO. 2008. p. 15–35.

[3] FAO. Applying spatial planning for promoting future aquaculture growth. Seventh session of the Sub-Committee on Fisheries (COFI), St Petersburg, Russian Federation; Discussion document: COFI:AQ/VII/2013/6; 2013 October 7–11; 2013.

[4] FAO. Aquaculture zoning, site selection and area management under the ecosystem approach to aquaculture. Policy brief; 2015.

[5] Cochrane K, De Young C, Soto D, Bahri T, editors. Climate change implications for fisheries and aquaculture: overview of current scientific knowledge. FAO Fisheries and Aquaculture Technical Paper. No. 530. Rome, FAO; 2009.

[6] Rosa R, Marques A, Nunes ML. Impact of climate change in Mediterranean aquaculture. Rev Aquacult. 2012; 4:163–177.

[7] Kapetsky JM, Aguilar-Manjarrez J. Geographic Information Systems, Remote Sensing and Mapping for the Development and Management of Marine Aquaculture. Tech. Pap. No. 458; FAO, Rome; 2007.

[8] Saitoh SI, Mugo R, Radiarta IN, Asaga S, Takahashi F, Hirawake T, et al Some operational uses of satellite remote sensing and marine GIS for sustainable fisheries and aquaculture. ICES J Mar Sci. 2011; 68:687–695.

[9] Brigolin D, Porporato EMD, Prioli G, Pastres R. Making space for shellfish farming along the Adriatic coast. ICES J Mar Sci. 2017, 74:1540–1551.

[10] Añel JA, (2011) The importance of reviewing the code, Communications of the ACM, 54 (5), doi: 10.1145/1941487.1941502

[11] FAO Fishstat J, Version 3.01 Aquaculture Production: Quantities 1950–2015. Available from: www.fao.org/fishery/statistics/software/fishstatj/en (accessed 16.03.2017).

[12] Brigolin D, Dal Maschio G, Rampazzo F, Giani M, Pastres R. An Individual-Based population dynamic model for estimating biomass yield and nutrient fluxes through an off-shore *Mytilus galloprovincialis* farm. Estuar Coast Shelf Sci. 2009; 82:365–376.

[13] Solidoro C, Pastres R, Melaku Canu D, Pellizzato M, Rossi R. Modelling the growth of *Tapes philippinarum* in the northern Adriatic lagoons. Mar Ecol Prog Ser. 2000; 199:137–148.

[14] Brigolin D, Pastres R, Tomassetti P, Porrello S. Modelling the biomass yield and the impact of seabream mariculture in the Adriatic and Tyrrhenian Seas (Italy). Aquac Int. 2010; 18:149–163.

[15] Brigolin D, Meccia VL, Venier C, Tomassetti P, Porrello S, Pastres R. Modelling biogeochemical fluxes across a Mediterranean fish cage farm. Aquac Environ Interact. 2014; 5(1):71–88.

[16] Bacher C, Gangnery A. Use of dynamic energy budget and individual based models to simulate the dynamics of cultivated oyster populations. J Sea Res. 2006; 56:140–156.

[17] Pastres R, Solidoro C, Cossarini G, Melaku Canu D, Dejak C. Managing the rearing of *Tapes philippinarum* in the lagoon of Venice: a decision support system. Ecol Model. 2001; 138:231–245.

[18] Rampazzo F, Berto D, Giani M, Brigolin D, Covelli S, Cacciatore F, et al Impact of mussel farm biodeposition on sediment biogeochemistry in the north-west Adriatic Sea. Estuar Coast Shelf Sci. 2013; 129:49–58.

[19] MiPAAF. Piano Strategico per l’acquacoltura in Italia, 2014–2020 Direzione Generale della Pesca e dell’Acquacoltura PEMAC; 2014.

[20] Jacob D, Petersen J, Eggert B, Alias A, Christensen OB, Bouwer LM, et al EURO-CORDEX: New high-resolution climate change projections for European impact research. Reg Envir Chang. 2014; 14:563 doi: 10.1007/s10113-013-0499-2

[21] Moss RH, Edmonds JA, Hibbard KA, Manning MR, Rose SK, van Vuuren DP, et al The next generation of scenarios for climate change research and assessment. Nature. 2010; 463:747–756. doi: 10.1038/nature08823 20148028 10.1038/nature08823

[22] Callier MD, Weise A, McKindsey CW, Desrosiers G. Sedimentation rates in a suspended mussel farm (Great-Entry Lagoon, Canada): biodeposit production and dispersion. Mar Ecol Prog Ser. 2006; 322:129–141.

[23] Zoppini A, Pettine M, Totti C, Puddu A, Artegiani A, Pagnotta R. Nutrients, standing crop and primary production in western coastal waters of the Adriatic Sea. Estuar Coast Shelf Sci. 1995; 41:493–513.

[24] Solidoro C, Bastianini M, Bandelj V, Codermatz R, Cossarini G, Canu DM, et al Current state, scales of variability, and trends of biogeochemical properties in the northern Adriatic Sea. J Geophys Res. 2009; 114(7):C07S91.

[25] Brigolin D, Lourguioui H, Taji MA, Venier C, Mangin A, Pastres R. Space allocation for coastal aquaculture in North Africa: data constraints, industry requirements and conservation issues. Ocean Coast Manag. 2015; 116:89–97.

