# Supplementary material for: An R package for simulating growth and organic wastage in aquaculture farms in response to environmental conditions and husbandry practices
Source: PLoS One. 2018 May 3;13(5):e0195732. doi: 10.1371/journal.pone.0195732 (PMC5933756; doi:10.1371/journal.pone.0195732)
Supplement: S1 File — Table A. Model state variables, forcings, and functional relationships of M. galloprovincialis–as in Brigolin et al. (2009). Table B. Functional expressions used in the individual growth models of D. labrax and S. aurata–as in Brigolin et al. (2010; 2014). Table C. Model state variables, forcings, and functional relationships of R. philippinarum—as in Solidoro et al. (2000). (PDF) [file pone.0195732.s001.pdf]

S1 File. Model equations.

An R package for simulating growth and organic wastage in aquaculture farms in response to environmental conditions and husbandry practices

Damiano Baldan[1], Erika Maria Diletta Porporato[2], Roberto Pastres[1,2], Daniele Brigolin[1,2*]

[1]Bluefarm S.r.l., Venezia Marghera, Italy

[2]Department of Environmental Sciences, Informatics and Statistics, Ca' Foscari University of Venice, Venezia Mestre, Italy.

[*] Corresponding author

E-mail: brigo@unive.it (DBr)

Table A. Model state variables, forcings, and functional relationships of *Mytilus galloprovincialis* – as in Brigolin et al. (2009)

**Prognostic state variables**<br>W_b : somatic dry weight [g]<br>R: gonadic dry weight [g]<br><br>**Diagnostic state variables**<br>W_d : dry weight of the mussel [g]<br>W_f: wet weight of the mussel [g]<br>W_{tot} : total weight of the mussel, including the shell [g]<br>L: length of the shell [cm]	**1. Growth equations**<br>$\frac{dW_b}{dt} = (1-k)\cdot\frac{(A\text{-}C)}{\varepsilon_B}$ (1)<br>$\frac{dR}{dt} = k\cdot\frac{(A\text{-}C)}{\varepsilon_R}$ (2)
	**2. Computation of the available energy**<br>$Cp = chl2cp\cdot CHL$ (3)<br>$PHY = \frac{Cp}{\gamma}$ (4)<br>$DT = POC - Cp$ (5)
**Forcings**<br>T_w : water temperature [°C]<br>*POC*: Particulate Organic Carbon concentration [mgC l^{-1}]<br>*CHL*: Chlorophyll-a concentration [mg l^{-1}]<br>*PHY:* Phytoplankton concentration [mg l^{-1}]<br>*Cp*: Phytoplankton-C concentration [mgC l^{-1}]<br>*TSM*: Total Suspended Matter concentration [mg l^{-1}]<br>*POM*: Particulate Organic Matter concentration [mg l^{-1}]<br>*DT*: Organic detritus concentration [mg l^{-1}]<br><br>**Parameters**<br>*chl2cp*: Conversion factor from CHL to Cp [-]<br>γ: Conversion factor from Cp to Phy [-]<br>AE_{max} : Maximum Adsorption Efficiency [-]<br>K_s : Half-saturation constant for the AE [-]<br>T_{ma} : Maximum temperature for the anabolic processes [°C]<br>T_{oa} : Optimal temperature for the anabolic processes [°C]<br>β_a : Temperature exponent for the anabolism [1/°C]<br>Cr_{max} : Maximum Filtration rate [l/(day·gDW)]	**3. Functional expressions for net anabolism**<br>$f_a = \left(\frac{T_{ma}-T_w}{T_{ma}-T_{oa}}\right)^{\beta_a\cdot(T_{ma}-T_{oa})}\cdot e^{\beta_a\cdot(T_w-T_{oa})}$ (6)<br>$I = CR_{\max}\cdot f_a\cdot W_b^q\cdot\left(\varepsilon_{DT}\cdot DT+\varepsilon_{Phy}\cdot PHY\right)$ (7)<br>$Q = \frac{POM}{TSM}$ (8)<br>$AE = AE_{\max}\cdot\frac{Q}{Q+K_s}$ (9)<br>$E = AE\cdot I$ (10)<br>$A = (1-\alpha)\cdot E$ (11)
	**4. Functional expressions for fasting catabolism**<br>$f_c = \left(\frac{T_{mc}-T_w}{T_{mc}-T_{oc}}\right)^{\beta_c\cdot(T_{mc}-T_{oc})}\cdot e^{\beta_c\cdot(T_w-T_{oc})}$ (12)<br>$C = R_{\max}\cdot f_c\cdot\varepsilon_{O_2}\cdot W_b$ (13)
	**5. Reproduction events**<br>$R(t=spawn_1)=R(t=spawn_2)=0$ (14)
	**6. System output**<br>$W_d = W_b + R$ (15)<br>$W_f = a_f\cdot W_d$ (16)<br>$W_{tot} = a_{tot}\cdot W_d$ (17)

q: Weight exponent for filtration [-]<br>ε_{DT} : Energy content of detritus [J/mg]<br>ε_{PHY} : Energy content of phytoplankton [J/mg]<br>α: Feeding catabolism [-]<br>A_{max} : Maximum energy ingestion rate for a 1 g mussel [J/(g·day)]<br>T_{mc} : Maximum temperature for the catabolic processes [°C]<br>T_{oc} : Optimal temperature for the catabolic processes [°C]<br>β_c : Temperature exponent for the catabolism [1/°C]<br>R_{max} : Maximum respiration rate [mgO2/(gDW·day)]<br>ε_{O2} : Energy consumed by the respiration of 1g of oxygen [J/mgO2]<br>k: Energy fraction invested in reproduction [-]<br>ε_B : Somatic tissue energy content [J/g]<br>ε_R : Gonadic tissue energy content [J/g]<br>a_f: Dry weight-wet weight conversion coefficient [-]<br>a_{tot} : Dry weight-total (including shell) weight conversion coefficient [-]<br>a_L : Weight-length conversion coefficient [mm/mg^{bL}]<br>b_L : Shape-coefficient for the weight-length conversion [-]	$L = a_L \cdot W_d^{\,b_L}$ (18)

Table B. Functional expressions used in the individual growth models of *Sparus aurata* and *Dicentrarchus labrax* – as in Brigolin et al. (2010; 2014)

State variable:

w: fresh weight [g]

Growth equation:

$$\frac{dw}{dt} = \left(\frac{A - C}{\varepsilon_T} \right);$$

A: net anabolism [J day^{-1}]

C: fasting catabolism [J day^{-1}]

ε_T: energy content of somatic tissue [kJ g^{-1}]

Forcings:

T_w : water temperature [°C]

R: amount of food provided by the farmer per individual [g day^{-1}]

C_P : % of proteins in the ingested food

C_C : % of carbohydrates in the ingested food

C_L : % of lipids in the ingested food

Functional expressions for net anabolism

$$I = I_{max} \cdot H(Tw) \cdot w^m$$

I: daily ingestion rate [g day^{-1}]

I_{max}: maximum ingestion rate [g day^{-1} g^{-m}]

m: weight exponent for the anabolism

F: faeces production [g day^{-1}]

$$\begin{cases} I = R & , when\ I \geq R \\ I = 0 & , when\ T < T_a \end{cases}$$

$$A = (1-\alpha) \cdot I \cdot [C_P \cdot \varepsilon_P \cdot \beta_P + C_C \cdot \varepsilon_C \cdot \beta_C + C_L \cdot \varepsilon_L \cdot \beta_L]$$

$$F = I \cdot [C_P \cdot (1-\beta_P) + C_C \cdot (1-\beta_C) + C_L \cdot (1-\beta_L)]$$

α: feeding catabolism coefficient

β_P, β_C, β_L: assimilation coefficient for protein, carbohydrate and lipid

ε_P, ε_C, ε_L: energy content of protein, carbohydrate and lipid [kJ g^{-1}]

2. Functional expressions for fasting catabolism

$$C = \varepsilon_{O2} \cdot k_0 \cdot K(T_w) \cdot w^n$$

ε_{O2}: energy consumed by the respiration of 1 g of oxygen [kJ g^{-1}]

k_0: fasting catabolism at 0°C [day^{-1} g^{-n}]

n: weight exponent for the catabolism

$$H(T_w) = \left(\frac{T_m - T_w}{T_m - T_o}\right)^{b(T_m - T_o)} \cdot e^{b(T_w - T_o)}$$

b: shape coefficient for the $H(T_w)$ function

T_o: optimal temperature [°C]

T_m: maximum lethal temperature [°C]

$$K(T_w) = e^{pk \cdot T_w}$$

pk: temperature coefficient for the fasting catabolism [$°C^{-1}$]

O: daily respiration rate [day^{-1}]

$Ex_{P,N}$: daily dissolved N,P excretion rates [day^{-1}]

$$O = k_0 \cdot K(T_w) \cdot w^n$$

$$Ex_N = O \cdot k_{N:O}$$

$$Ex_P = O \cdot k_{P:O}$$

**3. Wasted feed (W)**<br>*W*: uneaten feed [g day^{-1}]<br>$\begin{cases} W = R - I & ,when\ R \geq I \\ W = 0 & ,when\ R < I \end{cases}$

Table C. Model state variables, forcings, and functional relationships of *Ruditapes philippinarum* - as in Solidoro et al. (2000)

W_w : wet weight [g]<br>W_d : dry weight [g]<br>*L*: length of the shell [mm]<br>b: coeff. of allometric equation relating w_d to w_w<br>a: coeff. of allometric equation relating w_w to L	**Isometric relation**<br>$W = aL^3$<br>**Allometric relation**<br>$w_d = bw_w^p$
T: water temperature [°C]<br>F: Food concentration in water [µg chl-a l^{-3}]<br>G_{wmax} : Max. growth rate on a wet weight basis [gww$^{1/3}$ day^{-1}]<br>r_{wmax} : Max. respiration rate on a wet weight basis [day^{-1}]	**Growth equations**<br>if E > E*, which is equivalent to F > F*<br>$\frac{dL}{dt} = G_{L\max} f_{gT}(T) f_{gF}(F) - r_{L\max} f_{rT}(T) L$<br>$\frac{dw_w}{dt} = G_{w\max} f_{gT}(T) w_w^{2/3} - r_{w\max} f_{rT}(T) w_w$

G_{dmax} : Max. growth rate on a dry weight basis [gdw$^{0.265}$ day^{-1}]<br>r_{dmax} : Max. respiration rate on a length basis [day^{-1}]<br>G_{Lmax} : Max. growth rate on a length basis [mm day^{-1}]<br>r_{Lmax} : Max. respiration rate on a length basis [day^{-1}]<br>p: Coeff. of allometric growth equation relating w_d to w_w [-]<br>q: Coeff. of allometric filter velocity [-]<br>T_{mG} : Max. temperature for growth [°C]<br>T_{oG} : Optimal temperature for growth [°C]<br>T_{mr} : Max. temperature for respiration [°C]<br>T_{ov} : Optimal temperature for filtration [°C]<br>ε_F : Energetic content food [J µg chl-a^{-1}]<br>ε_T : Energetic content of<br>*Ruditapes philippinarum* [J g dw^{-1}]	$\frac{dw_d}{dt} = G_{d\max} f_{gT}(T) w_d^{(1-1/3p)} - r_{d\max} f_{rT}(T) w_d$<br>if E ≤ E*, which is equivalent to F ≤ F*<br>$\frac{dL}{dt} = \frac{F}{F^*} G_{L\max} f_{gT}(T) f_{gF}(F) - r_{L\max} f_{rT}(T) L$<br>$\frac{dw_w}{dt} = \frac{F}{F^*} G_{w\max} f_{gT}(T) w_w^{2/3} - r_{w\max} f_{rT}(T) w_w$<br>$\frac{dw_d}{dt} = G_{d\max} f_{gT}(T) w_d^{(1-1/3p)} - r_{d\max} f_{rT}(T) w_d$<br>$= F V_f f_v(T) w_d^q \frac{\varepsilon_F}{\varepsilon_T} - r_{d\max} f_{rT}(T) w_d$<br>with<br>$G_{d\max} = p G_{w\max} b^{(1/3p)} = p G_{r\max} 3 a^{1/3} b^{(1/3p)}$<br>$r_{d\max} = p r_{w\max} = 3 r p_{r\max}$<br>Threshold below which growth is food limited<br>$F^* = \frac{G_{d\max} f_{gT}(T) w_d^{(1-1/3p)} \varepsilon_T}{V_f f_v(T) w_d^q \varepsilon_F}$<br>Functional response of temperature for growth, respiration and filtration
	$f_{gT}(T) = (\frac{T_{mG} - T}{T_{mG} - T_{oG}})^{\beta_G (T_{mG} - T_{oG})} e^{\beta_G (T - T_{oG})}$<br>$f_{tT}(T) = (\frac{T_{mr} - T}{T_{mr} - T_{or}})^{\beta_r (T_{mr} - T_{or})} e^{\beta_r (T - T_{or})}$<br>$f_v(T) = (\frac{T_{mv} - T}{T_{mv} - T_{ov}})^{\beta_v (T_{mv} - T_{ov})} e^{\beta_v (T - T_{ov})}$
