# Supplementary material for: An R package for simulating growth and organic wastage in aquaculture farms in response to environmental conditions and husbandry practices
Source: PLoS One. 2018 May 3;13(5):e0195732. doi: 10.1371/journal.pone.0195732 (PMC5933756; doi:10.1371/journal.pone.0195732)
Supplement: S2 File — Table A. Parameters used in the Mytilus galloprovincialis growth model. Table B. Parameters used in the Sparus aurata (SA) and Dicentrarchus labrax (DL) growth models. Table C. Parameters used in the Ruditapes philippinarum growth model. (PDF) [file pone.0195732.s002.pdf]

S2 File. Model parameters.

An R package for simulating growth and organic wastage in aquaculture farms in response to environmental conditions and husbandry practices

Damiano Baldan[1], Erika Maria Diletta Porporato[2], Roberto Pastres[1,2], Daniele Brigolin[1,2*]

[1]Bluefarm S.r.l., Venezia Marghera, Italy

[2]Department of Environmental Sciences, Informatics and Statistics, Ca' Foscari University of Venice, Venezia Mestre, Italy.

[*] Corresponding author

E-mail: brigo@unive.it (DBr)

Table A. Parameters used in the *Mytilus galloprovincialis* growth model.

Parameter	Description	Value	Unit
chl2cp	Conversion factor from *CHL* to *Cp*	50	mgC mg^{-1}
γ	Conversion factor from *Cp* to *Phy*	0.38	mgC $mgDW^{-1}$
AE_{max}	Maximum Adsorption Efficiency	0.8	-
K_s	Half-saturation constant for the AE	0.157	-
T_{ma}	Maximum temperature for the anabolic processes	29	°C
T_{oa}	Optimal temperature for the anabolic processes	20	°C
β_a	Temperature exponent for the anabolism	0.21	$°C^{-1}$
Cr_{max}	Maximum Filtration rate	107	$l\ (day \cdot gDW)^{-1}$
q	Weight exponent for filtration	0.53	-
ε_{DT}	Energy content of detritus	12.5	J mgC^{-1}
ε_{PHY}	Energy content of phytoplankton	61.8	J $mgDW^{-1}$
α	Feeding catabolism	0.092	-
A_{max}	Maximum energy ingested from a mussel of 1 g	430	$J\ (gDW \cdot day)^{-1}$
T_{mc}	Maximum temperature for the catabolic processes	29	°C
T_{oc}	Optimal temperature for the catabolic processes	20	°C
β_c	Temperature exponent for the catabolism	0.25	$°C^{-1}$
R_{max}	Maximum respiration rate	14.5	mgO_2 $(gDW \cdot day)^{-1}$
ε_{O2}	Energy consumed by the respiration of 1g of oxygen	14	J mgO_2^{-1}
N:O	$N\text{-}NH_4^+$ excreted over $O\text{-}O_2$ consumed conversion coefficient	0.013	-
k	Energy fraction invested in reproduction	0.3	-
ε_B	Somatic tissue energy content	20893	J g^{-1}
ε_R	Reproductive tissue energy content	28302	J g^{-1}
a_f	Dry weight-wet weight conversion coefficient	7.0	-
a_L	Weight-length conversion coefficient	6.7	mm mg^{-bL}
b_L	Shape-coefficient for the weight-length conversion	0.29	-
$spawn_1$	Autumn-winter spawning	15-Dec	day
$spawn_2$	Spring-summer spawning	15-Jun	day

Table B. Parameters used in the *Sparus aurata* (SA) and *Dicentrarchus labrax* (DL) growth models.

Parameter	Description	Value SA	Value DL	Unit
I_{max}	Maximum ingestion rate	0.09	0.05	g food g fish^{-m} day^{-1}
α	Feeding catabolism coefficient	0.3	0.3	-
β_P	Assimilation coefficient for protein	0.85	0.88	-
β_C	Assimilation coefficient for carbohydrate	0.5	0.84	-
β_L	Assimilation coefficient for lipid	0.95	0.97	-
ε_P	Energy content of protein	23.6	23.6	kJ g^{-1}
ε_C	Energy content of carbohydrate	17.2	17.2	kJ g^{-1}
ε_L	Energy content of lipid	36.2	36.2	kJ g^{-1}
ε_{O2}	Energy consumed by the respiration of 1g of oxygen	13.6	13.4	kJ g_{O2}^{-1}
ε_T	Energy content of somatic tissue	9.9	9.18	kJ g^{-1}
pk	Temperature coefficient for the fasting catabolism	0.06	0.05	°C^{-1}
k_0	Fasting catabolism at 0°C	0.00072	0.00069	g 0_2 g fish^{-1} day^{-1}
m	Weight exponent for the anabolism	0.6	0.6	-
n	Weight exponent for the catabolism	1	1	-
b	Shape coefficient for the $H(T_w)$ function	0.2	0.23	-
T_o	Optimal temperature	25.0	22.0	°C
T_m	Maximum lethal temperature	32.9	32.0	°C
T_a	Lowest feeding temperature	12.0	7.0	°C

Table C. Parameters used in the *Ruditapes philippinarum* growth model.

Variable	Explanation	Value	Unit
G_{wmax}	Max. growth rate on a wet weight basis	0.0312	$gww^{1/3}$ day^{-1}
r_{wmax}	Max. respiration rate on a wet weight bas	0.0081	day^{-1}
G_{dmax}	Max. growth rate on a dry weight basis	0.01456	$gdw^{0.265}$ day^{-1}
r_{dmax}	Max. respiration rate on a dry weight basis	0.0102	day^{-1}
G_{Lmax}	Max. growth rate on a length basis	0.16	mm day^{-1}
r_{Lmax}	Max. respiration rate on a length basis	0.0027	day^{-1}
m	Coeff. of Ursin equation	2/3	
n	Coeff. of Ursin equation	1	
a	Coeff. of allometric equation relating w_w to L	0.00026	gdw mm^{-3}
b	Coeff. of allometric equation relating w_d to w_w	0.0234	
p	Coeff. of allometric equation relating w_d to w_w	1.26	
q	Coeff. of allometric filter velocity	0.32	
T_{mG}	Max. temperature for growth	32.0	°C
T_{oG}	Optimal temperature for growth	22.7	°C
β_G	Coeff. of temperature growth	0.2	$°C^{-1}$
T_{mr}	Max. temperature for respiration	35.0	°C
T_{or}	Optimal temperature for respiration	20.5	°C
β_r	Coeff. of temperature respiration	0.17	$°C^{-1}$
T_{mv}	Max. temperature for filtration	32.0	°C
T_{ov}	Optimal temperature for filtration	22.7	°C
β_v	Coeff. of temperature filtration	0.2	$°C^{-1}$
ε_F	Energetic content food	4.7	J μg^{-1} chl *a*
ε_T	Energetic content of *Ruditapes philippinarum*	19200	J gdw^{-1}
V_f	Max. filtration rate	2.3	l day^{-1} $(gdw^q)^{-1}$
