# Supplementary material for: An R package for simulating growth and organic wastage in aquaculture farms in response to environmental conditions and husbandry practices
Source: PLoS One. 2018 May 3;13(5):e0195732. doi: 10.1371/journal.pone.0195732 (PMC5933756; doi:10.1371/journal.pone.0195732)
Supplement: S1 Appendix — (PDF) [file pone.0195732.s003.pdf]

S1 Appendix. R code to run mechanistic model spatially explicit

An R package for simulating growth and organic wastage in aquaculture farms in response to environmental conditions and husbandry practices

Damiano Baldan[1], Erika Maria Diletta Porporato[2], Roberto Pastres[1,2], Daniele Brigolin[1,2*]

[1]Bluefarm S.r.l., Venezia Marghera, Italy

[2]Department of Environmental Sciences, Informatics and Statistics, Ca' Foscari University of Venice, Venezia Mestre, Italy.

[*] Corresponding author

E-mail: brigo@unive.it (DBr)

```
library(raster)
library(rgdal)
library(sp)
library(ncdf4)

setwd ("…..")
sst <- stack ("sst.nc")
chl <- stack ("chl.nc")
pixel_sst <- rasterToPoints (sst)
pixel_sst_t <- t (pixel_sst)
coord <- pixel_sst[,1:2]
sst <- pixel_sst_t[-c(1,2),]
sst <- sst-273.15 # If the SST data are in Kelvin
pixel_chla<- rasterToPoints (chl)
pixel_chla_t <- t (pixel_chla)
chl <- pixel_chla_t[-c (1,2),]

##### RUN MECHANISTIC MODEL ####
library(RAC)
userpath <- "….."
RAC::Mussel_ind_skeleton (userpath)   ###Warning: Overwrite existing files!
forcings <- Mussel_ind_dataloader (userpath)

output=i
for (i in 1: ncol (sst) {
forcings [[2]] <- sst[,i]
forcings[[4]] <- chl[,i]
output[i] <- Mussel_ind_main (userpath, forcings)
out [ , i] <- unlist(c(t(output[[1]])))
daysout[ , i] <- unlist(c(t(output[[7]])))}

L <- matrix (out, ncol = nrow(sst))
L <- t (L)
daysout <- t (daysout)
```

```
L_spatial <- cbind (coord[ ,2:3], L[,])
days_spatial<- cbind (coord[ ,2:3], daysout [,])

####### FINAL LENGHT MAP ##########
coordinates (L_spatial) <- ~x+y
proj4string (L_spatial) = CRS ("+proj=longlat +datum=WGS84")
gridded (L_spatial) = TRUE
Final_lenght = raster (L_spatial)
projection (Final_lenght) = CRS ("+proj=longlat +datum=WGS84")
plot (Final_lenght)
Lenght_brick_days <- brick(Final_lenght)
Lenght_brick_days <- setZ (Lenght_brick_days, as.Date(“YYYY-m-d”) + 0: nrow (sst))

########### DAYS TO COMMERCIAL SIZE #######
coordinates (days_spatial) <- ~x+y
proj4string (days_spatial) = CRS ("+proj=longlat +datum=WGS84")
gridded (days_spatial) = TRUE
days_spatial = raster (days_spatial)
projection (days_spatial) = CRS ("+proj=longlat +datum=WGS84")
plot (days_spatial)
days_brick_days <- brick(days_spatial)
days_brick_days <- setZ (days_brick_days, as.Date(“YYYY-m-d”) + 0: nrow(sst))


####### STORE IN NETCDF ##########
writeRaster (Lenght_brick_days, "Daily_length_mussels.nc", format="CDF", varname="length",
varunit= "cm", longname="Length of Mytilus galloprovincialis estimated through the R RAC
package developed by Baldan et al.", zname="time", zunit="day")

writeRaster (days_brick_days, "Days_to reach_CS_mussels.nc", format="CDF", varname="days",
varunit= "day", longname="Days to reach the commercial size required by Mytilus
galloprovincialis estimated through the R RAC package developed by Baldan et al.",
zname="time", zunit="day")
```
